# 

**Published:** 1857-09

**Authors:** 


					﻿CONTENTS OF VOL. XI.
A Separating Forcep, Valuable for Extracting Dens Sapient®..........■............. 253
A Few Notes concerning the Progress of Dental Surgery............................. 361
Abscess—Diagnosis of Tooth-ache................................................... 224
“ Advice to those who use Amalgam”................................................ 220
American Dental Convention—Review............................................... 347
American Dental Convention, Proceedings of,........................................ 28
Amalgam. By E. Townsend........................................................... 440
Anaesthetics Agents, A Safe Substitute for them................................... 439
Bending Plates.................................................................... 354
Boston Meeting.................................................................... 103
Biographical Sketch of Prof. Shotwell............................................. 106
Cheoplasty, “ Novice”............................................................. 159
Cheoplasty-•.................................................................. 349,374
Chemistry, Dental of the Mouth. By Geo. Watt...................................... 10
Dental Convention of Northern Ohio................................................ 171
Dental Societies................................................................. 275
Dental Progress-•>................................................................ 220
Dr. Blakesley’s Method of Filling Teeth........................................... 352
Editorial..............................................................112, 229,355,470
Extracting Teeth.................................................................. 217
Filling Teeth. By J. Taft....................................................5, 137,233
Functions of the Nervous System............................................... 168,	368
Filling Labial Surfaces of Upper Incisors. By A. J.’Volck, D. D. S................ 104
Gladstone’s Lectures.......................................................... 425,	428
Improved Plates. By Dr. J. Winter................................................. 348
Interesting Case of Ilemicrania. By J. L. Levison, D. D. S........................ 156
Introductory Lecture to the General Course of Mechanical Dentistry. By J. Richard-
son, D. D. S.................................................................. 121
Irregularity—Correction. By Dr. W. W. Allport.................................... 21
Little Troubles with a Plate Tooth. By A. Berry, D. D. S........................ 168
Miscellaneous..........................................................104,204,349,450
Method of Promptly Relieving Facial and Dental Neuralgias......................... 210
Minutes of the Ohio Dental College Association.................................... 316
Mississippi Valley Association of Dental Surgeons—Minutes......................... 319
“	“	“	“	“ Discussions............................ 326
Moulding Sand—To Improve it....................................................... 350
New Method of Plating............................................................. 222
New York Dental Association—Minutes and Discussions............................... 292
Nitrate of Silver in Tooth-ache................................................... 216
“ Novice,” Noticed................................................................ 260
On Clasps. By C. W. Spalding, D. D. S............................................ 433
On Gelatinized Chloroform......................................................... 211
Paine on Treating Gold Filings, etc................................................ 20
Partial Sets of Artificial Teeth. By H. R. Smith................................... 33
Popular Essays on Dentistry. By II. C. Preebles................................... 431
Professional Character. By Dr. F. S. Slosson...................................... 373
Professional Personalities........................................................ 247
Proceedings of Societies................................................28, 171, 292, 38j**
Report on Dental Progress.......................................................... 282
Remarks upon the Teeth of the Natives of India..................................... 251
Richardson on Malposition........................................................... 19
Third Annual Meeting of the American Dental Convention............................. 145
“	“	“	“ Michigan Dental Association................................ 385
The Poverty of Dentists............................................................ 257
The Ethics of the Dental Profession. By B. P. Aydelott, D. D....................... 265
Treatment of Exposed Pulp. By. W. R. Lilly......................................... 350
Ulcerative Stomatitis.............................................................. 225
Valerianate of Ammonia......................................................... 214,	349
Valedictory Address. By C. B. Chapman.............................................. 237
“	“	“ Piggot..................................................... 435
“Vent Filling. By W. H. Atkinson................................................... 371
Vicarious Menstruation following the Extraction of a Tooth. By J. Richardson,
D. D. S........................................................................ 377
Western Pental Society............................................................. 351
ELEVENTH VOLUME
OF THE
DENTAL REGISTER OF THE WEST.
EDITED BY
J. T2kJ?T	cA Gr. ’W-A-'TT.
TERMS:.............................S3	PER ANNUM, IN ADVANCE.
Advertisements Inserted as Follows :
One page, per annum..........................................$25	CO
One-half page, per annum..................................... 15	00
Quarter page, per annum...................................... 10	00
Shorter periods than one year, by special arrangement.
Kj?Contributions to the pages of the Register respectfully solicited.
Exchanges and correspondents will please address, “ Dental Register,” or “Taft & Watt,
C incinnati, Ohio.”
				

